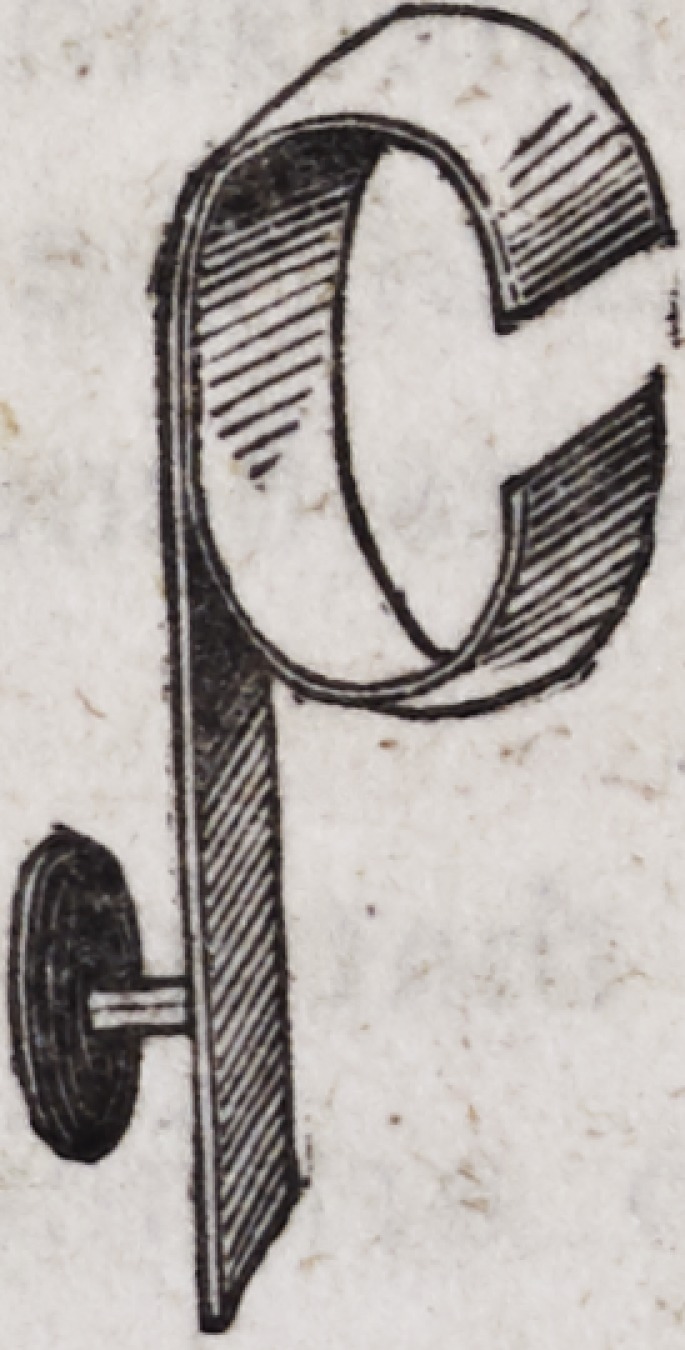# A Treatise on Mechanical Dentistry

**Published:** 1843-03

**Authors:** Solyman Brown


### ARTICLE II.

*A Treatise on Mechanical Dentistry.* By Solyman Brown, M. D., D. D. S.

(Continued from page 97.)

### CHAPTER V.

### *On Double Sets with Springs.*

98. In the foregoing chapters the student has been instructed in all parts of the process of constructing both upper and lower sets of artificial, mineral teeth, insomuch that he is now prepared to study the approved methods by which they are retained in their proper positions in the mouth, as the component parts of entire double sets.

99 Nearly a century ago, Pierre Fauchard, a dental surgeon in Paris, France, published a series of plates representing the methods then in vogue for securing double sets of teeth in their proper position. These pieces were at that time made exclusively of ivory, and contained no metal in their construction except the springs.

The following is an exact copy of one of Fauchard's engravings, executed A. D., 1746.

The same intelligent and industrious author gives a drawing of an upper set retained in its place by means of a metallic fixture connected with the lower teeth which are supposed to be in their natural position while the upper set has been destroyed by disease.

100. The disadvantages of both these machines, will be manifest to the experienced dentist, and can hardly fail to suggest themselves to the most casual observer. So true is this, that when compared with more modern contrivances, and especially with the improved double sets of the present day, which are constructed of incorruptible materials throughout, they deserve to be remembered chiefly for their antiquity. This may serve to illustrate to dental students of the present age, the great advantage which they enjoy in having a more elevated starting point from which to commence their race of professional enterprise. Happy indeed may they esteem themselves at the end of their career, if they shall be able to point out to their successors in dental practice, an equal amount of improvement resulting from their ingenious and persevering exertions, as has marked the progress of those who have gone before them.

101. I shall not deem it expedient to trace minutely the steps between the age of Fauchard and our own times, by which the profession has been led progressively by untiring effort to the present improved methods of sustaining and constructing entire double sets of artificial teeth. Suffice it to say that much labour and no little ingenuity have been bestowed upon the subject not only by dentists who have gone before us, but by many who still remain the ornaments of their profession.

If any thing still more perfect be the subject of benevolent anticipation in relation to this particular department of dental practice, it only proves that the gratitude of the members of the profession, as well as of community at large, may still be secured by the fortunate individuals who accomplish these improvements, as it has been by those who have elevated our art to its present distinction.

102. The simplest of the methods of connecting and sustaining double sets of teeth, at present pursued, may be described as follows. A metallic spiral spring of gold, alloyed to sixteen carats by equal parts of silver and virgin copper, drawn into wire of the size of a common sewing needle, is thus formed, in private practice, where the artist is not provided with a machine constructed for this special purpose. Let the wire be firmly secured between two blocks of soft wood held in the jaws of a common bench vice. Let one end of the wire be grasped by a hand-vice in conjunction with about six inches of hardened steel wire of the size of a common knitting needle. By revolving the steel wire as it rests on the end of the wooden blocks, by means of the hand-vice, the gold wire will be wound closely around the steel, with a firmness proportioned to the pressure of the bench vice upon the blocks of wood.

A few experiments will teach the necessary pressure upon the wooden blocks in order to force the gold wire around the steel rod with sufficient power. As one of the most important properties of this spiral spring, is its elasticity, care must be taken that in drawing down the gold wire to its proper size, it must not be annealed or softened towards the close of the operation of drawing it. Let it pass through four or five holes of the draw plate without being exposed to heat. It follows of course that the spring itself must not be heated by the blow-pipe or otherwise after being wound into its spiral form.

A better method of forming these springs, is to employ a simple machine constructed for the purpose by the principal manufacturers of dental apparatus. In our large cities the profession are generally supplied by their gold-beater, or some persons who manufacture those springs for the market.

103. One inch and a quarter or an inch and a half is about the common length of these springs when completed, to which when the eyelets are attached, it will give an inch and three-fourths, or two inches and a fourth, as the entire distance between the centres of motion.

The eyelets, as represented above, are made of thick gold plate, and are much the most uniform and beautiful when cut from the plate by means of a punch moved by machinery, which forms them at a stroke. A screw may be cut on the end of the eyelet which enters the spring, or the end may be left square and introduced with such force as to render it sufficiently secure. When a screw is to be cut on the eyelet—let the end on which the screw is to be formed consist of a piece of gold wire which may afterward be soldered to the flat portion of the eyelet as follows:

Eyelets constructed in this manner, with two distinct pieces of metal, are much the strongest and on this account are generally preferred.

104. The pivots, by means of which these eyelets are attached to the two principal plates on which the teeth rest, are constructed as follows:—a circular plate of thick gold, about one-fourth of an inch in diameter, having a hole in the centre, may be formed neatly by means of a file, or struck by a punch and machinery constructed for the purpose. A gold-wire pin of the size of a large knitting needle is then introduced into the central orifice and firmly soldered.

A is a circular disk of gold plate, B a gold pin of the size required, C both the preceding joined together: D the whole when soldered and filed into form. The heads of these pivots may be turned and polished in a small lathe, when such an instrument is at hand.

105. A gold standard is next required for the purpose of attaching these pins to the main plate on which the teeth rest. This is made of strong gold plate of the following form, having a curvature designed to embrace the anterior surface of the second bicuspis.

These standards are firmly soldered, first to the pins or pivots before described, and afterwards to the principal plate, as represented in the following cut.

106. In those cases where the standards are attached to the plate before the teeth, it will be generally expedient to make the standards flat instead of curved, inasmuch as it will be very difficult to determine the precise position which the second bicuspis will assume; and indeed it frequently happens that it may be advisable to place the standard nearer to the first bicuspis, or to the first large molar, in order to insure a just balance of the work. Sometimes also it happens that the standards on the upper plate are required to be placed either forward or backward of those on the lower plate, for the purpose of meeting some peculiarity of conformation in the mouth to which it belongs. The determination of these nice points will not fail to call into constant requisition the mechanical ingenuity of the operator, of which he will never find himself in possession of superabundance in encountering, with success, the difficulties and emergencies of his art.

107. As there is always some danger that an accident may happen to one of the eyelets before described, such as breaking off the point; a practice has been adopted by some dentists, to split the eyelet longitudinally through the centre of the pivot hole, in order that it may be removed or replaced at pleasure. I have found this stratagem to be of practical utility on many occasions, and have given a visible representation of it in the following cut;

A represents the eyelet when opened for the purpose of inserting or removing it; B represents it when closed upon the pivot. This section is most conveniently made by means of a very fine saw.

108. A still simpler and more easily practicable although a less workman-like method of attaching the springs to the pivots is represented in the following diagrams.

This eyelet is made of a piece of gold or platina wire, the latter being the least liable to break, either round or square, and in many instances, especially when required to be made in haste, it may answer a very valuable purpose.

109. An entire double set of artificial mineral teeth, when fitted with spiral springs and all the necessary appurtenances, as just described, assumes the following appearance when ready for insertion in the mouth.

As these beautiful and useful contrivances for restoring articucate speech, personal beauty and healthy appropriation of solid nutriment, are now constructed of imperishable materials, so far as any forms of matter are imperishable, it ought not to be forgotten, either by the skilful dentist or his fortunate patient, that the present age alone, of all the ages of the world since the commencement of the historical era, has the honour or the advantage of appropriating to itself this invaluable invention.

110. A few dental practitioners of my acquaintance have made the experiment of dispensing with the standards altogether, by using bicuspides having pivot holes passing through them from front to back, to receive the pivots already described.

But it is found by experiment that several objections to this procedure have urged its abandonment; the chief of which is that the mineral teeth as they are at present constituted, are frequently broken by the pivots.

111. A serious objection has always existed, to the practice of confining the pivots on which the spiral springs move to any definite and fixed position, inasmuch as it is sometimes found to be exceedingly difficult, if not quite impossible for the most expert and experienced artist to determine beforehand what are the exact centres of attachment for the fixed points of the springs. To obviate this acknowledged difficulty many ingenious contrivances have been adopted, among which the following appears to have been the most successful in practice.—Instead of an upright standard, such as has been already described for the purpose of attaching the springs to the plate, a thin plate of gold is formed into the following shape, being five-eighths of an inch in length, one-sixth of an inch in width, and one-twelfth of an inch in depth.

This slide socket may be formed with facility on an iron or steel mandril of the shape and size required. The plate from which it is formed should be about half the thickness of common dentists' gold plate. This socket should be soldered to the principal plate in such a manner as to extend from the first bicuspis to the first large molar, as is seen in the following diagram.

This pin which secures the eyelet, is then furnished with a square or oblong plate of gold just broad and thick enough to play closely as a slide in the fixture just described. The pivot and its slide when firmly united by soldering, have the following appearance.

This slide when working in its box appears as in the following cut.

This apparatus can be made to remain in any desired position by pressing down the free edges of the plate composing the slide-box, so that they shall bear with the necessary degree of force upon the slide.

It is evident that with this contrivance the spiral spring can be so adjusted, by moving the slide backwards and forwards, as to bring them to bear properly both upon the upper and under set of teeth, for the purpose of keeping them exactly in their just position.

I do not intend to inculcate the opinion that an apparatus like that here described is always, or even generally either necessary or desirable. On the contrary I am convinced that in a majority of cases where entire double sets are required, the simple mode of attachment described in sections 103, 104, and 105, is greatly preferable to any more complicated apparatus. It will be found in practice, that, in nine cases out of ten, the stationary pivots when adjusted with judgment guided by experience, answer every important purpose, and at the same time, avoid all the hazards of more laboured contrivances.

And even in those instances when the sliding-box is adapted to meet the peculiarities of individual cases, it is necessary to furnish only one of the plates with this contrivance, which in general should be the lower one. The best opportunity of arranging the sliding-box to avoid interference with the muscles of the cheeks, must in most cases determine the question whether it be attached to the upper or the lower set. Still, as I have already said, there is generally the most room below.

112. The sliding-box just described, has been adopted for the purpose of accomplishing two important objects; first, to institute a just balance of support for the upper set, so that neither the forward nor posterior part shall be unduly abandoned to the action of gravitation and thus left to fall away from the gums and palate:—and, secondly, that the spiral springs may be so adjusted as not to impinge unnecessarily upon the sensitive and irritable tissues of either jaw. The former of these objects cannot be attained effectually without either employing the sliding-box, or adjusting the fixed pivot in such a manner as to balance the weight to be supported;—and this latter alternative may sometimes compel the artist to re-construct his work after much labor and expense. The second object, on the contrary, namely, adjusting the spiral springs in such a manner as to avoid unnecessary irritation either above or below, has been attained by several distinct methods of procedure, which as they are equally ingenious and instructive I shall proceed particularly to describe.

113. The first of these contrivances to which I shall allude, is the horizontal cylinder employed to prevent the spiral spring from falling too low upon the solid portions of the lower jaw, or rising too high against those of the upper. This cylinder which is of gold plate about a fourth of an inch in length, and of such a diameter as just to receive the end of the spiral spring, is soldered to the principal plate in the following manner:—

It is obvious to the slightest inspection that the spiral spring at the point A will not descend to the point B, so long as it is inserted firmly at its extremity in the horizontal cylinder, as it would do if it were left to play freely upon a pivot, as seen in section 109 of this chapter. In this manner a local irritation may be avoided in cases of peculiar conformation of the parts, when a double set could not be endured of the simple construction in ordinary use.

When the spiral spring does not fit the cylinder with sufficient exactness to secure it against the accident of being too easily withdrawn in the act of mastication, and also for the purpose of excluding particles of food from the barrel of the spiral spring, a gold screw may be inserted into the end of the spring, which will remove the difficulties to which we have referred. In some cases it may be necessary to employ only one of these barrels to a double set when the formation of the jaw requires no more.

114. A second contrivance to prevent the springs from irritating the parts by pressing too hard in a perpendicular or vertical direction, is to arm the head of the pivot with a flange or protuberance extending inwards and placed in such a position as to arrest the motion of the eylet when approaching the spot to be protected. For the purpose of rendering this contrivance intelligible to every reader, I shall give a somewhat enlarged view of the parts, so that they may be distinctly seen and understood. And, in the first place, the head of the pivot instead of being an exact circle, must be furnished with a flange or projection, from a part of its circumference, as in the following figure:—

This flange must be turned or bent at right angles with the plane of the circle so that it shall assume the following form:—

When the pivot has been attached to this head, and the eyelet placed upon its pivot, the pivot must be soldered to the standard in such a position that when the standard shall have been attached to the principal plate, the flange of the head shall arrest the eyelet when in such a position as to prevent the spring from impinging upon the jaw. In this way the same object will be attained as by the horizontal cylinder mentioned in section 113, and at less expense of labour and material. The following diagram is intended to illustrate the manner in which this flange operates to arrest the motion of the spring at any point chosen by the artist. This is an elegant artifice which does great credit to the inventor.

In most instances it will be found expedient to insert a washer of gold between the eyelet and the standard for the purpose of preventing the spring from crowding too hard upon the teeth. The thickness of this washer must be determined by the relative situation of the teeth beside which the spring plays.

115. While treating of the construction of double sets, it becomes necessary to instruct the student in what manner to proceed when the lower set of the natural teeth of the patient, are nearly all still in their places, especially six or eight of the front teeth, after all those of the upper jaw have been removed by disease. In this case the artificial arrangement is denominated a double set, and the dentist is compelled to charge the same price for it as when all the natural teeth of both jaws are wholly wanting, because the work is quite as difficult if not more so than in entire double sets. In a few instances only, when the formation of the mouth is favourable to such a course, the plate may be struck from a single piece of gold in the same manner as when all the teeth are to be restored. In cases of this kind it must be presumed that the portion of the plate which passes immediately behind the natural teeth, can be of such width as to possess the strength required so as not to be liable to be easily bent or broken.

116. The difficulty of finding a sufficient space between the incisor and the sublingual muscles, in ordinary mouths, to allow of forming the whole plate of one piece of gold, renders it necessary in a majority of cases to pursue the following method.

Strike two distinct lateral plates for those separate portions of the alveolar arch on which artificial teeth are to rest, and fix them firmly in their places upon a plaster cast of the jaw, by means of plaster and sand. Provide a semi-cylinder of gold, or a half round wire about a fourth or three-eighths of an inch in breadth, and having bent it exactly to conform to the posterior surface of the natural teeth and the gum behind them, file and fashion the ends of this wire so as to adapt it neatly to the surfaces of the two lateral plates. Fix this strap firmly in its place by means of small iron wire passing either around or through the plaster cast. Then after covering all parts of the work with sanded plaster, excepting the ends of the strap and the adjacent parts of the plates, solder the parts thoroughly together. When the half round wire cannot be procured, the strap may be made of a thick plate of gold alloyed with platina, which has great strength and elasticity; or two plates of common gold may be soldered together after having been bent to exactly the shape required and bound in three or four places by fine iron wire. Even when the strap is constructed of half round wire, it would be desirable when convenient, that it be composed of the alloy of platina and gold.

The following is the form which such a structure will assume when prepared for the attachment of the standards and springs which connect it with the upper set.

It is never desirable to attempt to adapt this strap either to the forms of the posterior surfaces of the teeth behind which it passes, or to the corrugations of the gums on which it may repose, inasmuch as all such pieces are subject to slight movements, both laterally and downwards, which would render a nice adaptation to the teeth and gums worse than useless. In those instances in which the strap passes directly behind the teeth, it is always better to give it a general curvature, so as just to touch the teeth without penetrating into the interstitial depressions:—and when the strap falls below the necks of the teeth, it should rest gently on the gum and muscular tissues beneath, without pressing upon them with unnecessary force.

117. It sometimes happens that an upper set of teeth must be sustained by springs, when no artificial teeth are needed in the lower arch, inasmuch as there are some few mouths to which an upper plate cannot be successfully adapted on the principle of atmospheric pressure. Although such cases are very rare, I am anxious that the dental student shall know how to encounter these anomalies with confidence and success.

Let us suppose then that an individual has lost all the teeth of the upper maxillary arch, and none in the lower jaw. The intelligent dental operator will prepare his upper set as in all other cases, and provide the springs, eyelets and pivots. Instead of a standard to be placed in front of an artificial second bicuspis, or first large molar, let a thimble be fitted to one or the other of those teeth, as the circumstances of the case may require. To this thimble the pivot must be soldered, so that the whole arrangement will assume the following appearance.

These thimbles may have flanges extending to the grinding surfaces of the teeth to which they are attached, in order to prevent them from being forced with violence upon the gums by the action of the springs.

118. In case the particular tooth to which the thimble is attached does not occupy the exact position required for the pivot, a plate may be soldered to the thimble in the direction of its radius, of any desired length, as follows:—

119. As I have before remarked, many other modes of accomplishing the objects thus attained have been adopted by our professional brethren, among whom there has not been wanting that mechanical ingenuity which the perplexing anomalies of the art require.

But I have given what I believe to be the simplest and the best known methods of constructing entire double sets of artificial, mineral teeth. Should any member of the profession be in possession of better methods than those which I have described; and should they have the disposition to communicate them to the students of the dental art, through the pages of the American Journal and Library of Dental Science, there can be little doubt that they will receive, as they will merit, the grateful acknowledgments of their brethren.

[TO BE CONTINUED.]

## Figures and Tables

**Figure f1:**
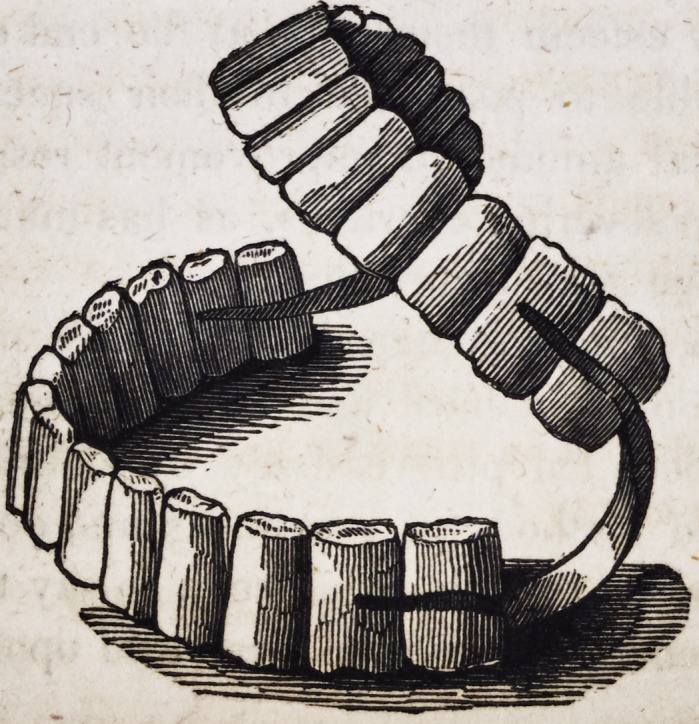


**Figure f2:**
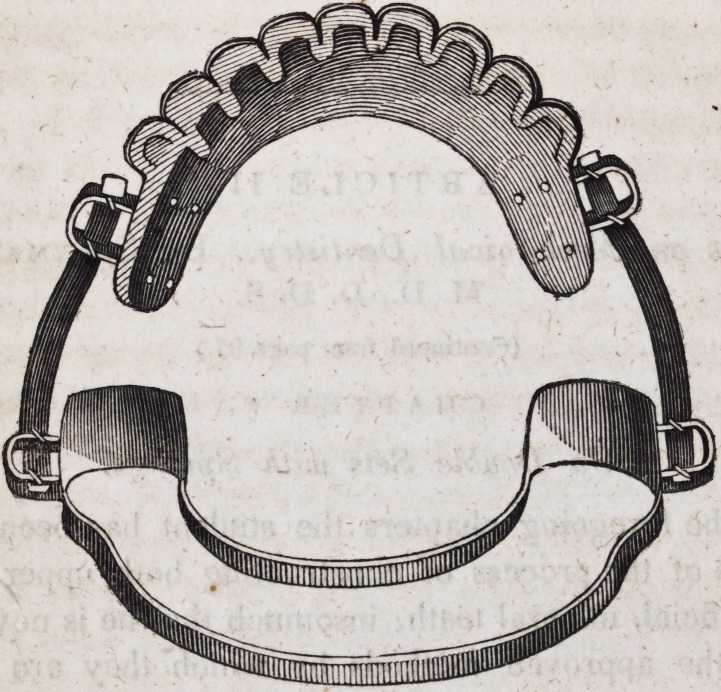


**Figure f3:**
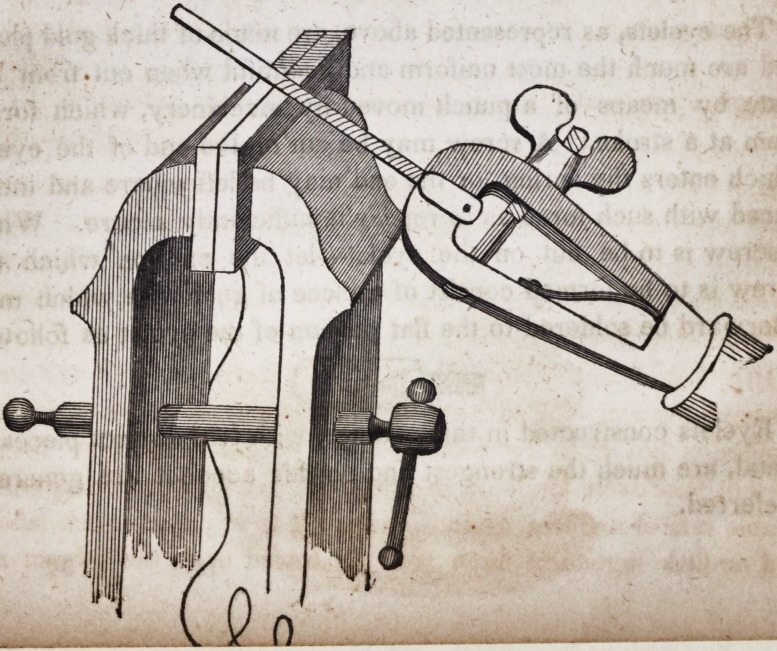


**Figure f4:**
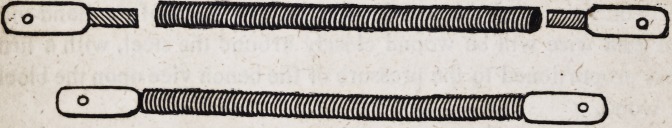


**Figure f5:**
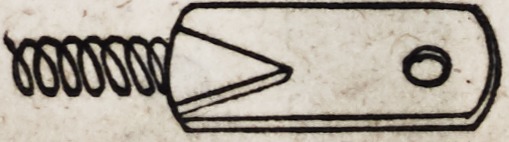


**Figure f6:**
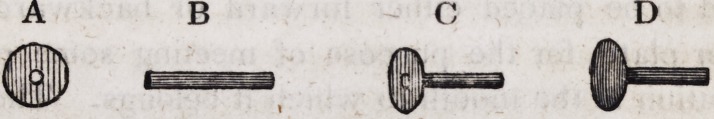


**Figure f7:**
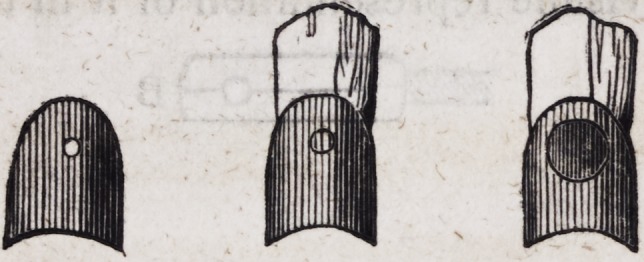


**Figure f8:**
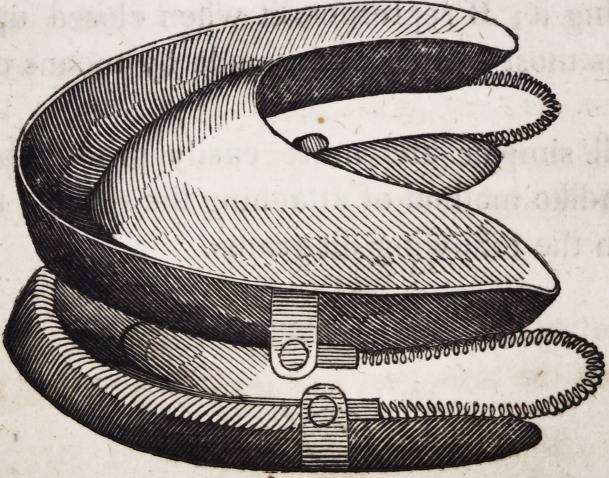


**Figure f9:**
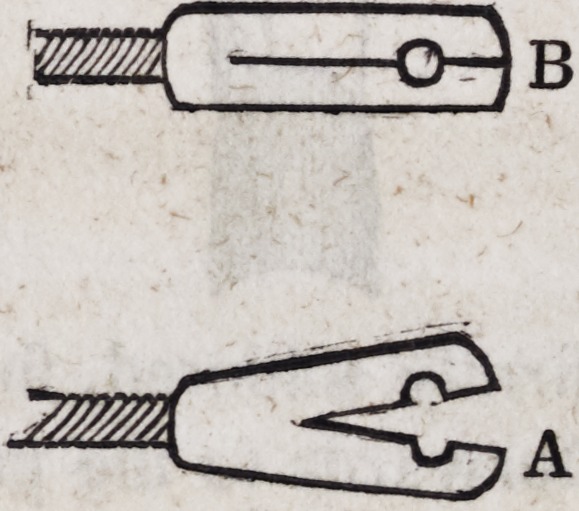


**Figure f10:**
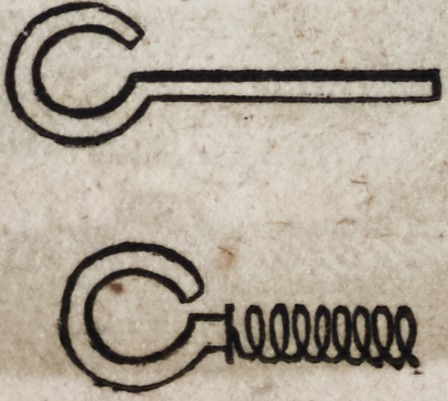


**Figure f11:**
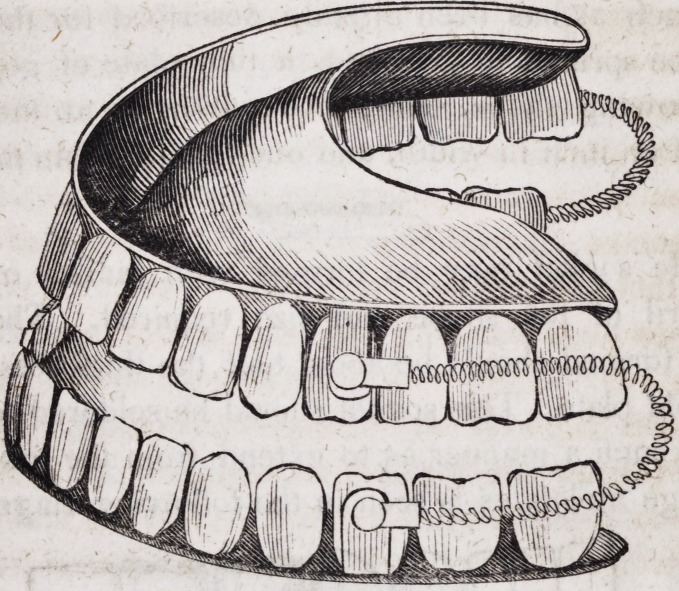


**Figure f12:**
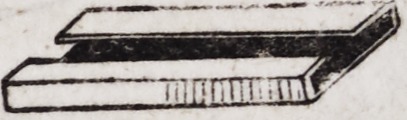


**Figure f13:**
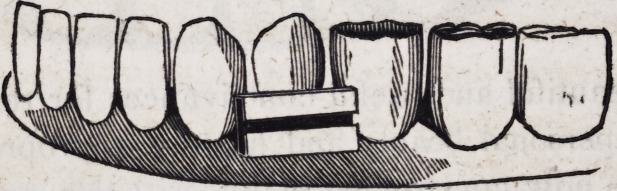


**Figure f14:**
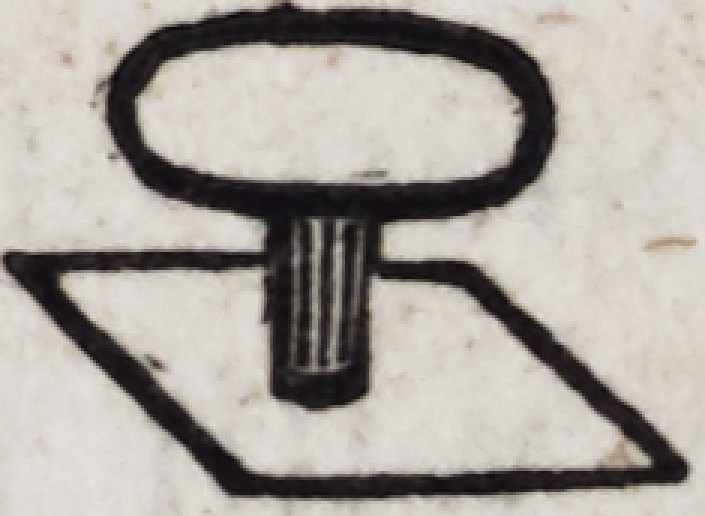


**Figure f15:**
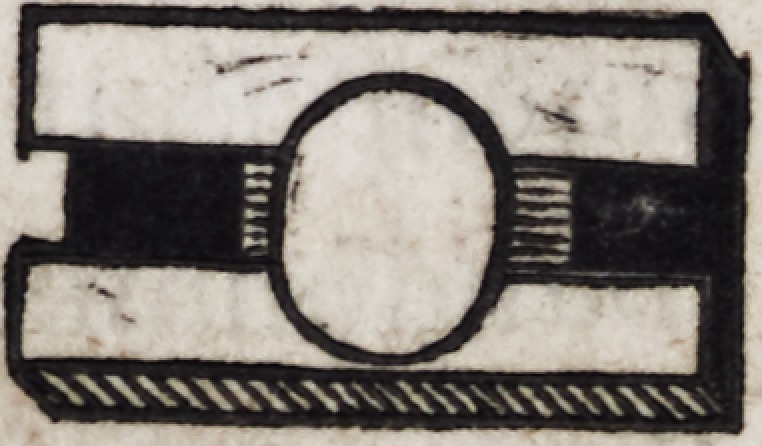


**Figure f16:**
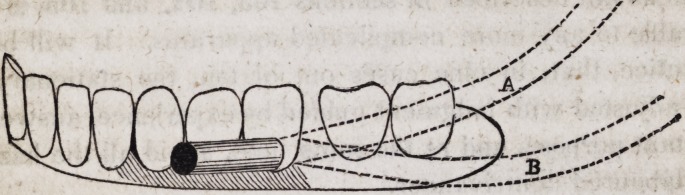


**Figure f17:**
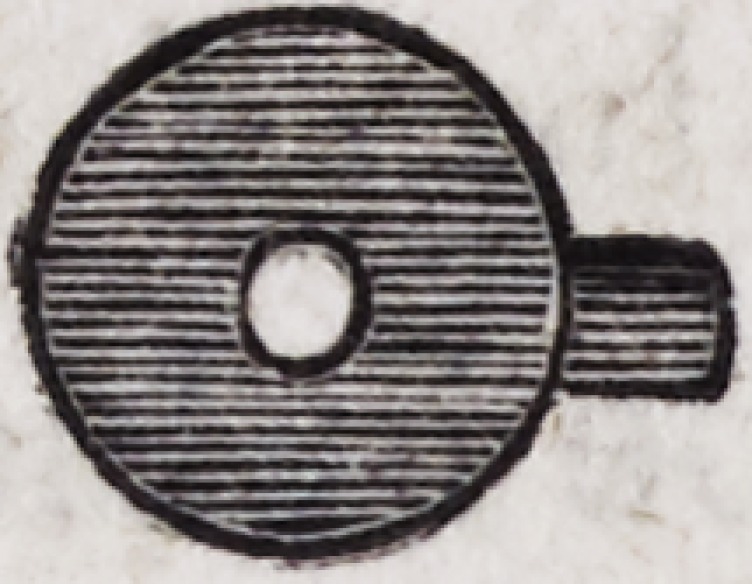


**Figure f18:**
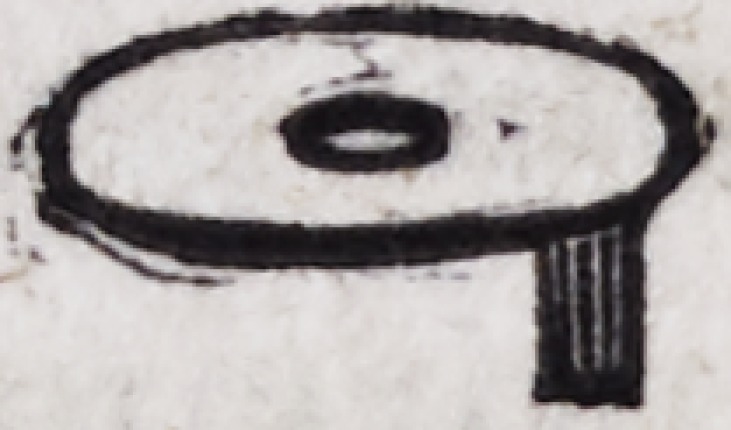


**Figure f19:**
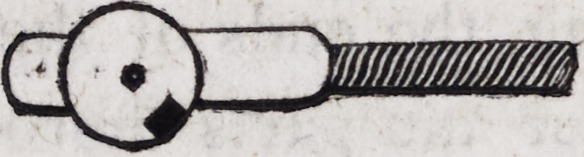


**Figure f20:**
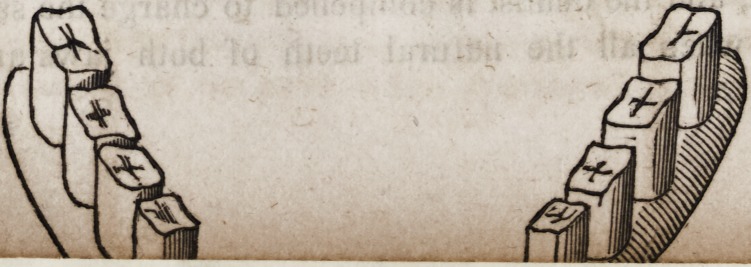


**Figure f21:**
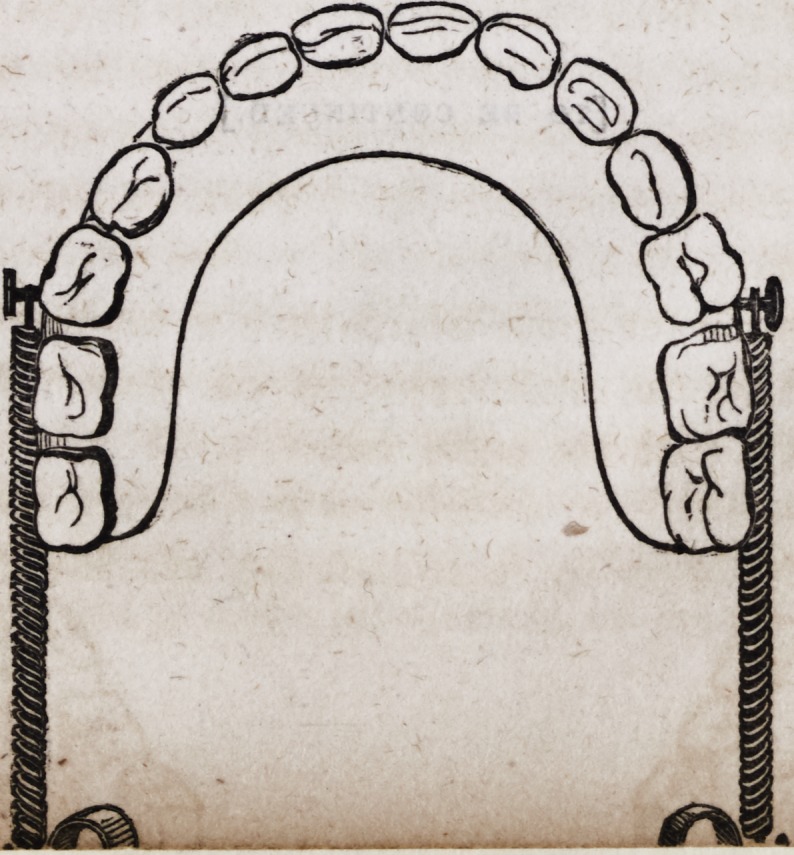


**Figure f22:**